# Dual-Action NO Delivery from One Mixed Metal Metal–Organic Framework

**DOI:** 10.1021/acs.inorgchem.4c05125

**Published:** 2025-02-05

**Authors:** Russell M. Main, Aaron B. Naden, Morven J. Duncan, Russell E. Morris, Romy Ettlinger

**Affiliations:** † EaStCHEM School of Chemistry, University of St Andrews, Purdie Building, North Haugh, St Andrews KY16 9ST, U.K.; ‡ TUM School of Natural Sciences, Technical University of Munich, Lichtenbergstraße 4, 85748 Garching bei München, Germany

## Abstract

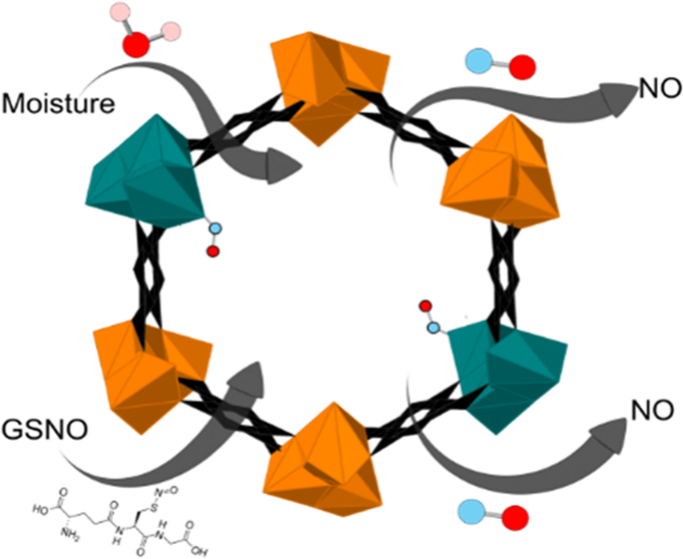

Owing to their varied and controllable reticular chemistry, metal–organic frameworks (MOFs) represent excellent, structurally precise material platforms for creating multifunctional devices. This flexibility allows the design of MOFs to feature two different delivery mechanisms for the medicinal gas nitric oxide (NO) within one structure: a rapid, kinetic release of preadsorbed NO and subsequent continuous, catalytic generation of NO on exposure to a suitable substrate. In our approach, this was realized by preparing mixed metal MOF-74 analogues (also known as CPO-27) combining two metals, namely, nickel and copper. The introduction of 10 atom % nickel into Cu-MOF-74 provides a material platform with a number of excellent properties: (i) a high capacity of NO can be released with a moisture-triggered mechanism; (ii) the available copper sites allow the (potentially indefinite) catalytic generation of NO in the presence of S-nitrosoglutathione (GSNO), a store of NO in the human body; and (iii) it features prolonged material stability on exposure to phosphates. This material therefore shows great promise as part of the next generation of multifunctional MOF-based medicinal devices.

## Abstract 2

We prepared mixed metal copper/nickel metal−organic frameworks of the MOF-74 family, capable of releasing nitric oxide (NO), an important biological signaling molecule, via two methods: rapid burst release from preadsorbed NO and continuous catalytic generation from S-Nitrosoglutathione (GSNO). This material shows great potential to be part of the next generation of medical devices.

## Introduction

Metal–organic frameworks (MOFs) are a well-studied class of porous materials.^1,2^ Made from metal ions linked by organic bridging units, there is already a vast library of MOF structures and topologies that have been synthesized.^3^ The use of reticular chemistry allows MOF structures and properties to be precisely fine-tuned by altering either the metal nodes and/or organic linker without affecting the topology.^4,5^ This can lead to a diverse range of improvements, from catalytic behavior^6^ to exceptional porosities.^7^ To date, MOFs have shown promise for many potential applications, including gas storage,^8,9^ catalysis,^10^ industrial separations,^11^ pollution capture,^12,13^ and medicine. They feature great capability for drug storage and release,^14,15^ targeted drug delivery,^16,17^ and the storage and triggered release of medicinal gases.

In this study, we selected the widely studied MOF-74 (also known as CPO-27), which has the highest density of open metal sites of any MOF, as a material platform.^18−20^ It consists of 1D-chains of M(II) ions (M = Mg, Ni, Co, and Cu···) bound together by 2,5-dihydroxylterephthlate linkers to produce a 3D porous framework with hexagonal channels. The accessible metal sites in the channels allow for a high storage capacity of polar gases, which favor chemisorption.^12,,22^ Additionally, through weaker interactions, gases can be physisorbed within the pores.^23,24^ The properties of MOF-74 can be altered through reticular chemistry, for instance, by changing the metal ions, with different gases preferring to bind different metals.^25^ This can be done with a wide degree of complexity, as shown by Wang et al., who demonstrated the successful incorporation of up to 10 different metal ions within a single framework (Mg, Ca, Sr, Ba, Mn, Fe, Co, Ni, Zn, and Cd).^26^ Such complex mixed metal systems can allow for multiple chemistries to be performed simultaneously, allowing almost enzymatic-like functionalities.^27^

In our previous work, we have reported on the use of Ni-MOF-74 (and other materials) to bind large quantities of nitric oxide (NO) to open metal sites. NO is a toxic gas, but it is also a neurotransmitter molecule known to have vasodilatory, antithrombotic, wound healing, and antimicrobial properties.^28−30^ The bound NO can be released/delivered by exchange with water, for instance, on exposure to moisture.^31,32^ This mechanism works for *M*-MOF-74 powders and when embedded within a polymer, i.e., as a mixed matrix membrane. Such composite materials have potential applications in medicine.

There are other approaches to produce NO. One is using the bioactive MOF, Cu(II)1,3,5-benzene-tris-triazole (CuBTTri, sum formula H_3_[(Cu_4_Cl)_3_(BTTri)_8_]). Its copper sites demonstrated the long-term catalytic generation of NO from the endogenous source S-nitrosoglutathione GSNO.^33^ This mechanism mimics that found in the body where copper-containing enzymes can break down GSNO and other nitrosoglutathiones.^34^

**Figure 1:**
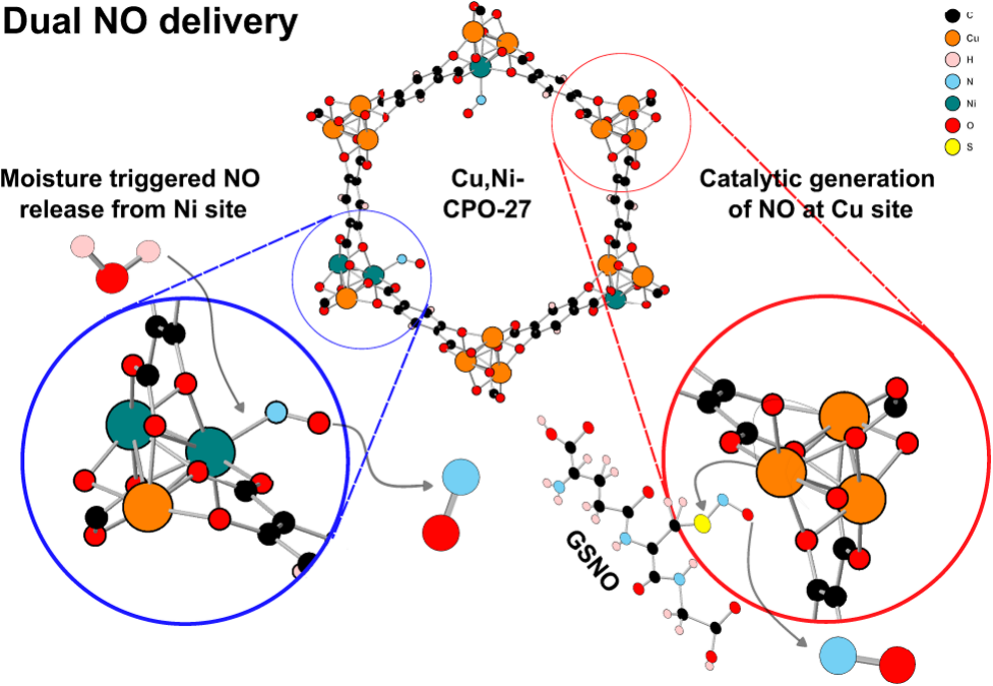
Schematic showing the dual NO delivery mechanisms that can be performed by Cu/Ni-MOF-74.

So far, the two mechanisms of gaseous NO release and NO generation via catalytic degradation of GSNO have been observed separately but never simultaneously from one framework. In this study, we shed light on mixed metal Cu/Ni-MOF-74 materials, which we show to be an ideal platform for both storing and releasing gaseous NO as well as catalytically producing it from GSNO (Figure 1).

## Results

### Material Preparation

**Figure 2:**
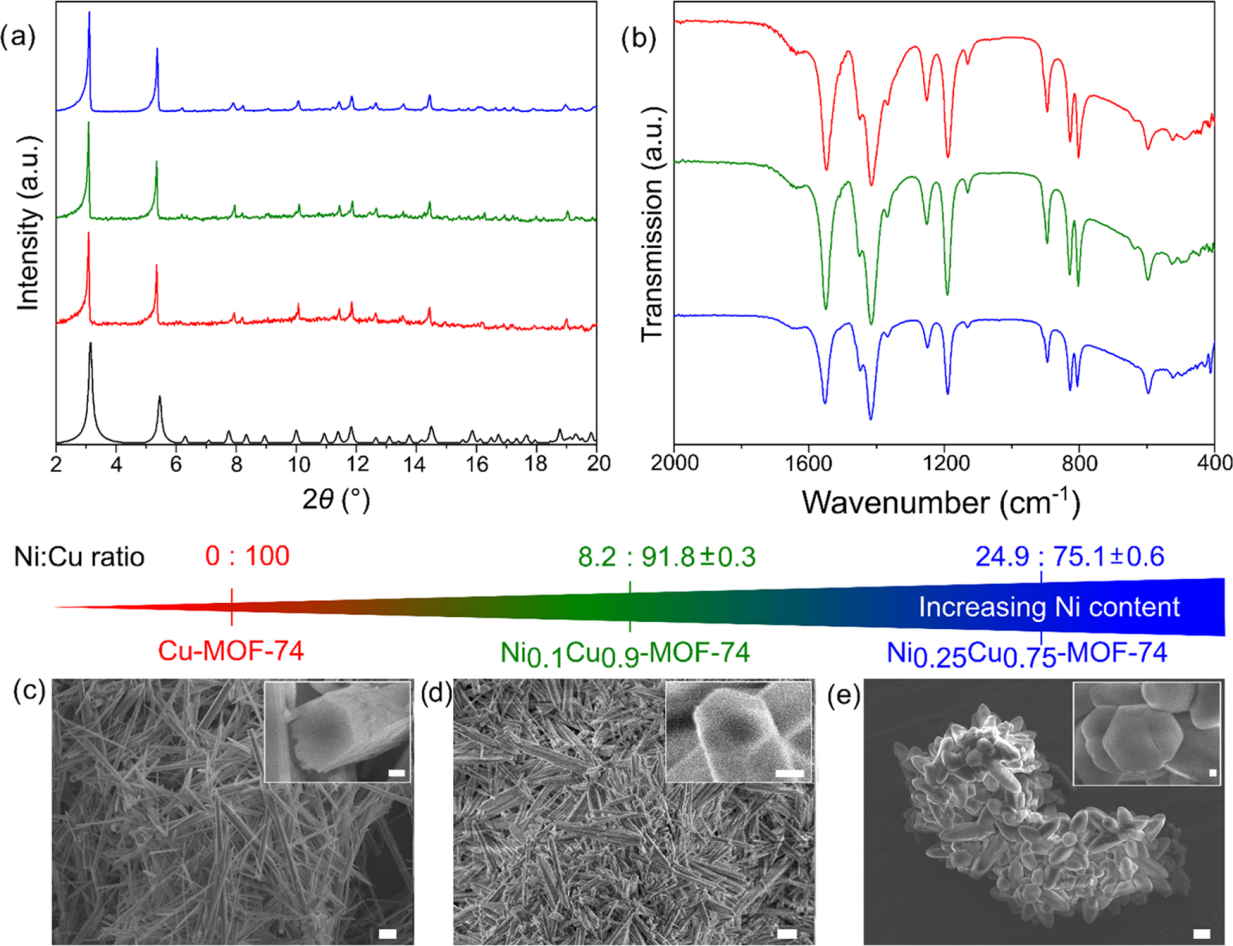
Characterization of Cu-MOF-74 (red), Ni_0.1_Cu_0.9_-MOF-74 (green), and Ni_0.25_Cu_0.75_-MOF-74 (blue): (a) PXRD patterns taken with Mo Kα radiation and compared to the calculated pattern (black); (b) FTIR spectra; and SEM images of (c) Cu-MOF-74, (d) Ni_0.1_Cu_0.9_-MOF-74, and (e) Ni_0.25_Cu_0.75_-MOF-74. Main scale bars: 2 μm; inset scale bars: 200 nm.

The selected MOF platform Cu-MOF-74 was synthesized following an adapted version of a literature procedure.^35^ The product was highly crystalline (Figure 2a, red) and showed a rod-like morphology (Figure 2c). By partial substitution of copper nitrate present during the MOF synthesis with different amounts of nickel nitrate, varying levels of nickel can be incorporated into the structure; the amounts of modulator and linker were fixed. Sample analysis using energy-dispersive X-ray spectroscopy (EDX) (Figures S1 and S2 and Tables S1 and S2) revealed that a reactant ratio of 1:1 Cu:Ni produced MOF-74 with 8 atom % nickel content (denoted hereafter as Ni_0.1_Cu_0.9_-MOF-74), whereas a ratio of 1:3 Cu:Ni yielded ≈25 atom % nickel content MOF-74 (denoted hereafter as Ni_0.25_Cu_0.75_-MOF-74). The lower level of nickel incorporation (compared to the amount added in the synthesis) into the MOF-74 structure might be attributed to the fact that this synthesis is carried out at 60 °C, whereas 130 °C is typically required for highly crystalline Ni-MOF-74.^36^ Reactions at temperatures above 60 °C were carried out; however, under these conditions, the products were contaminated with copper oxide impurities. Similar incorporation behavior has been observed previously in different MOFs and can be attributed to the lower lability of nickel compared to copper, exacerbated even further under these lower reaction temperatures.^31,37,38^ ICP-OES analysis was performed to further verify the obtained metal ratios in the bulk sample (Table S3). The amount of Ni in Ni_0.1_Cu_0.9_-MOF-74 was found to be the same as that obtained via EDX at 8 atom % nickel. ICP-OES analysis in Ni_0.25_Cu_0.75_-MOF-74 revealed that there was 17 atom % nickel compared to ∼25 atom % Ni using EDX. This slightly lower nickel content throughout the bulk of the material indicates the presence of defects and inhomogeneity within this analogue.

The purity of all three MOF-74 analogues was confirmed by powder X-ray diffraction (PXRD) (Figure 2a), as no additional reflections were observed compared to a calculated pattern. Fourier-transformed infrared spectroscopy (FTIR), in Figures 2b and S3, shows no additional bands compared with parent Cu-MOF-74, indicating the absence of any side products.

**Figure 3:**
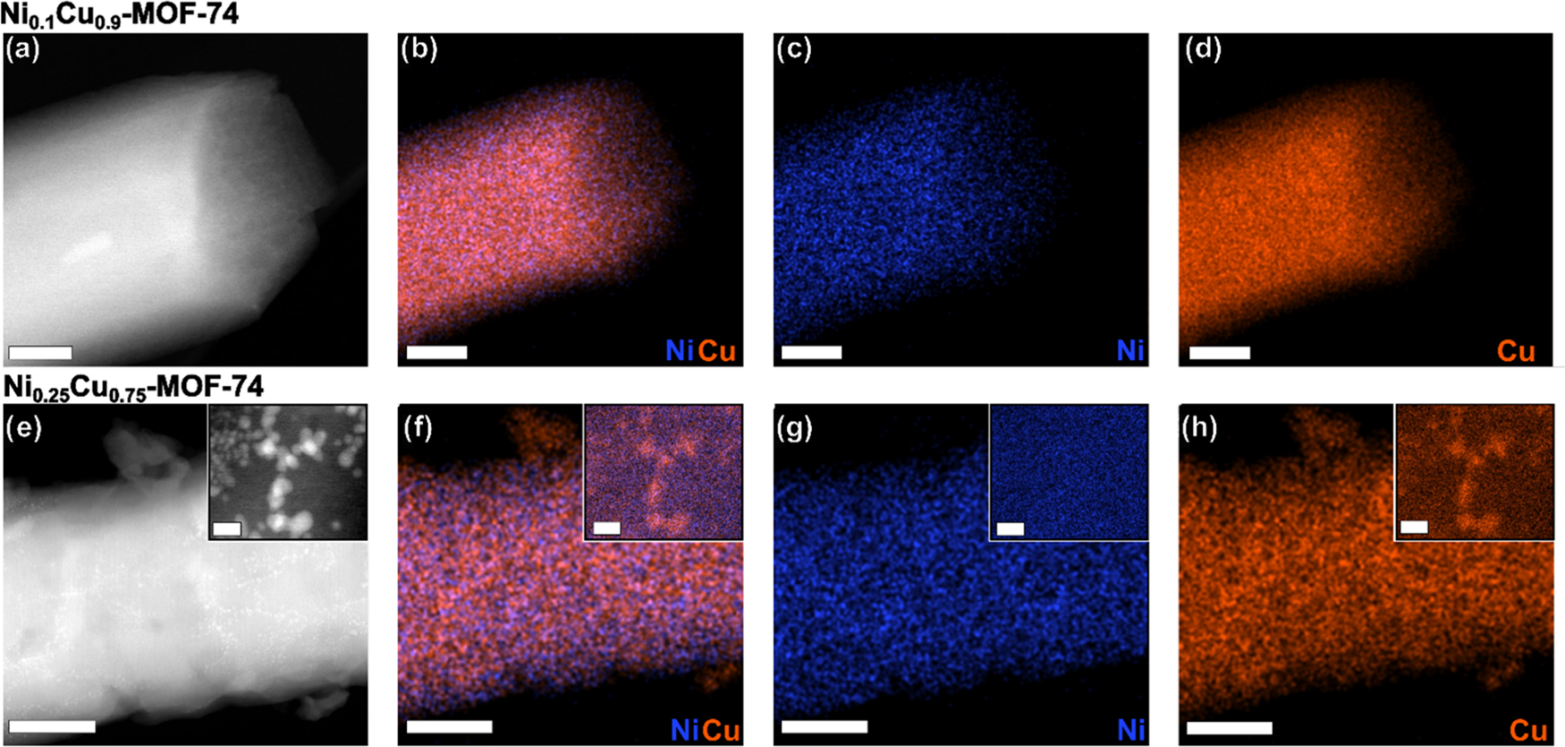
TEM images and elemental composition maps of Ni_0.1_Cu_0.9_-MOF-74 (top row) and Ni_0.25_Cu_0.75_-MOF-74 (bottom row): (a, e) HAADF images; (b, f) compositional overlap map of Cu (orange) and Ni (blue); (c, g) elemental map of Ni (blue); (d, h) elemental map of Cu (orange); Scale bars = 100 nm, 20 nm for the inset.

With an increasing concentration of nickel incorporation, the morphology of the samples subtly changed (Figure 2c–e). Cu-MOF-74 featured hexagonal rods, as did Ni_0.1_Cu_0.9_-MOF-74; however, these crystals were less uniform in size, with many being intergrown and exhibiting a more distorted hexagonal shape. Ni_0.25_Cu_0.75_-MOF-74 crystals displayed larger hexagonal lozenge-shaped crystals that were even more deformed and very intergrown. This continuous crystal deformation might be accounted for by nickel introducing strain into the crystal structure and/or reduction in copper nitrate concentration in the reaction mixture, slowing nucleation, thus altering crystal size and morphology. To visualize the metal distribution within the MOF structure, elemental composition maps were recorded (Figure 3). For Ni_0.1_Cu_0.9_-MOF-74, the individual compositional maps of nickel (blue) and copper (orange), as well as their overlay, confirmed a homogeneous distribution of both metals across the sample, indicating their random distribution within the 1D-chains (Figure 3a–d). The corresponding analysis of Ni_0.25_Cu_0.75_-MOF-74 revealed that these crystals generally also contained a homogeneous distribution of both metals (Figure 3e–h). The increasing amount of nickel, however, caused the formation of nanosized copper-rich agglomerates, which are visible as bright features throughout these crystals and on their surfaces (insets in Figure 3e–h). This finding may give an explanation as to the slight discrepancy in the nickel content measured by EDX (a surface-sensitive technique) and ICP-OES analysis (the bulk of the material). With the introduction of nickel into the MOF-74 structure, the thermal stability of the materials is also changed.

Thermal gravimetric analysis (Figure S4) shows that the mixed metal analogues degrade at a slightly lower temperature (∼10 °C) compared to the pure Cu-MOF-74. While the degradation profile of Ni_0.1_Cu_0.9_-MOF-74 is very similar to pure Cu-MOF-74, Ni_0.25_Cu_0.75_-MOF-74 shows an additional mass loss before the degradation temperature. This may be attributed to the increased number of defects in this material caused by the inclusion of the nanosized Cu agglomerates. To gain more insight into the framework integrity of the three different samples, N_2_ sorption isotherms were recorded at 77 K (Figure S5a). The analysis of the BET surface area for Cu-MOF-74 yielded a value of 1500 m^2^ g^–1^, which is in good agreement with the literature data, indicating the high quality of this sample. Ni_0.1_Cu_0.9_-MOF-74 had a slightly reduced surface area of 1116 m^2^ g^–1^. This reduction in the accessible specific surface area can be attributed to the intergrowth of the crystallites, inducing more defect-rich regions. Increasing the amount of nickel in the structure of Ni_0.25_Cu_0.75_-MOF-74 caused a further decrease in the specific surface area to 757 m^2^ g^–1^. This mainly reflects the progressively altered sample morphology as well as the presence of dense nanosized copper agglomerates, which cannot adsorb N_2_ and so will reduce the perceived surface area. Pore size distribution analysis (Figure S5b) reveals that both Cu-MOF-74 and Ni_0.1_Cu_0.9_-MOF-74 have pores of similar size, while Ni_0.25_Cu_0.75_-MOF-74 exhibits larger pores. This may be attributed to the higher degree of defects in the material caused by the inclusion of nanosized copper agglomerates.

### NO Delivery Performance

Following the successful synthesis of the three different porous Cu/Ni-MOF-74 analogues, the influence of the amount of nickel present on their ability to deliver NO gas was investigated. We recorded the release of NO under three different protocols: preadsorbed NO release studies, catalytic generation of NO from GSNO, and dual NO delivery analysis. Ni-MOF-74 has been shown previously to have excellent NO storage capacity but no ability to catalytically generate NO.^36,39^

#### Preadsorbed NO Release

**Figure 4:**
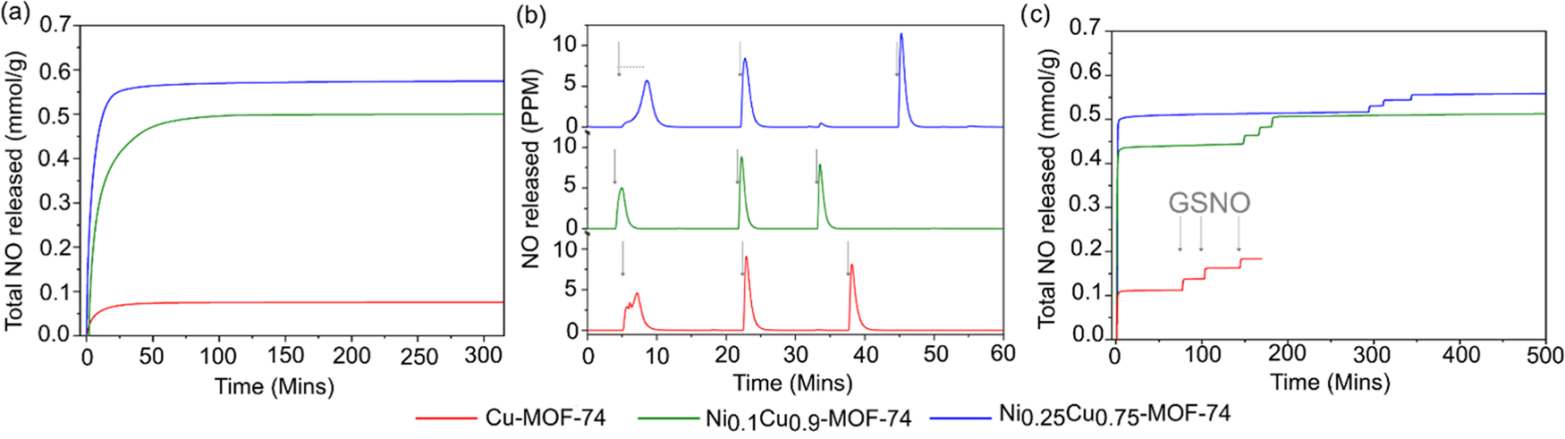
(a) Cumulative NO release profiles for the moisture-triggered mechanism of NO release (in 11% RH) for Cu-MOF-74 (red), Ni_0.1_Cu_0.9_-MOF-74 (green), and Ni_0.25_Cu_0.75_-MOF-74 (blue). (b) NO release profiles from the catalytic generation of NO from GSNO additions (gray arrows) in PBS for Cu-MOF-74 (red), Ni_0.1_Cu_0.9_-MOF-74 (green), and Ni_0.25_Cu_0.75_-MOF-74 (blue). (c) NO release profiles for dual mechanistic release of NO for Cu-MOF-74 (red), Ni_0.1_Cu_0.9_-MOF-74 (green), and Ni_0.25_Cu_0.75_-MOF-74 (blue).

To ensure maximal NO loading capacity, the three samples were first activated at 150 °C overnight (16 h) under vacuum, bathed in an atmosphere of NO gas (2 bar absolute pressure) for 1 h, evacuated, and backfilled with dry argon. The NO release capacity was recorded by flowing moist N_2_ gas (11% relative humidity [RH]) over the samples. The amount of NO released was measured using chemiluminescence.^39^Figure 4a shows the cumulated quantity of NO released from the metal sites via the moisture-triggered release mechanism from the Cu/Ni-MOF-74 analogues as a function of time. Pure Cu-MOF-74 (red) released the lowest quantity at 0.07 mmol of NO per gram. This is in agreement with the literature, as it has been shown that Cu-MOF-74 has a low affinity toward NO binding, which would reduce the amount available for release.^40^ However, upon the addition of only 10 atom % nickel (green), the amount of NO released is significantly increased to 0.50 mmol g^–1^, which corresponds to a more than 7-fold increase. The NO release capacity for Ni_0.25_Cu_0.75_-MOF-74 (blue) is slightly higher at 0.57 mmol g^–1^. These results show that incorporating only a small amount of nickel significantly increases the amount of NO released. Previous FTIR spectroscopy results and theoretical calculations suggest that Ni-MOF-74 has a stronger affinity for NO compared to Cu-MOF-74, which binds less NO due to a weaker binding interaction.^40,41^ However, no linear correlation between the amount of nickel incorporated into the MOF and the amount of NO released was observed. This lack of correlation may be attributable to the lower accessible specific surface area of Ni_0.25_Cu_0.75_-MOF-74 compared to Ni_0.1_Cu_0.9_-MOF-74 as well as to the altered particle size and inclusion of nanosized copper agglomerates.^36,42^ To achieve optimal NO release performance from a mixed metal MOF, a balance between chemical composition and framework integrity must therefore be considered.

#### Catalytic Generation of NO from GSNO

To test the capability of the different MOF-74 analogues to catalytically generate NO from GSNO, the samples were placed in phosphate-buffered saline (PBS) in a NO analyzer sample holder. Aliquots of GSNO were added to produce a concentration of 10 μM, similar to the concentration of S-nitrothiols found in human plasma.^43^ On GSNO addition, all three MOF-74 analogues were able to produce NO, as evidenced by the spikes in the NO release traces (Figure 4b). The Cu-MOF-74 (red) caused a rapid breakdown of GSNO as evidenced by the large spike of NO produced over 6 min (maximum NO release 5 ppm), resulting in a cumulated release of 0.014 ± 0.003 mmol g^–1^. When the quantity of NO fell below 5 ppb, additional PBS was added to ensure that all NO was released from the MOF, and subsequently, further aliquots of GSNO were added. These additions of GSNO were rapidly broken down within 4 min, as shown by the sharper NO spike in the trace. The cumulated release from both the second and third addition were 0.012 ± 0.002 mmol g^–1^, respectively. The broader first spike suggests the slower initial generation of NO, which may be due to the production of glutathione (GSH) required to produce the catalytic binding environment.^44^ The catalytic breakdown of GSNO was much quicker for Cu-MOF-74 than that seen with CuBTTri, where a slower release profile with a maximum of 40 ppb was obtained.^39^ The more rapid production of NO by Cu-MOF-74 can be explained by the higher amount of unsaturated copper sites available to perform the catalytic breakdown when compared to CuBTTri (4.6 copper sites/nm^3^ vs 1.9 copper sites/nm^3^, respectively). In addition, the hexagonal channels of MOF-74 may be more accessible to NO than the sodalite structure of CuBTTri.^33,45^ To further prove that the catalytic breakdown of GSNO can be assigned to the available copper sites in the MOF, an additional experiment without the MOF was performed. When GSNO was added to a vial containing only PBS (no MOF present), a small amount of NO was released, which can be attributed to the spontaneous oxidation of the GSNO (Figure S6). This release, however, is insignificant compared to the amount of NO released by the catalytic activity of the MOFs. Only the subsequent addition of CuCl_2_ could trigger catalytic oxidation of the previously added GSNO (Figure S6). However, the full breakdown of GSNO took 30 min. This 5 times faster rate of production of NO by Cu-MOF-74 highlights its excellent catalytic performance and suggests that the GSNO is reacting inside the pores of the framework. GSNO is small enough to fit inside the pores of MOF-74 in an end-on arrangement, where it can access the high density of copper sites.

The two mixed metal MOF-74 analogues can also rapidly generate NO, see Figure 4b. Ni_0.1_Cu_0.9_-MOF-74 (green) shows a release profile very similar to that of Cu-MOF-74 under the same experimental conditions (Figure 4b), releasing 0.015 ± 0.005 mmol g^–1^ of NO per aliquot of GSNO added. Although the initial spike has a slightly more Gaussian shape than that of Cu-MOF-74, the subsequent spikes are similarly narrower. This indicates that a similar induction period is occurring for both Ni_0.1_Cu_0.9_-MOF-74 and Cu-MOF-74. Ni_0.25_Cu_0.75_-MOF-74 (blue) can release 0.015 ± 0.004 mmol g^–1^ of NO per aliquot of GSNO added, similar to the case for the other two MOF-74 analogues. However, this trace shows that the initial generation of NO by Ni_0.25_Cu_0.75_-MOF-74 is slower, as indicated by a time lag from the addition of the GSNO to the onset of the NO release spike (horizontal dotted line in Figure 4b, blue). This longer induction period may be explained by the lower specific surface area or the nanosized copper agglomerates slowing down the GSH binding.

By analyzing the full width at half-maximum (fwhm) values for the second and third GSNO additions, an estimate of the relative kinetics for the production of NO from the three MOF-74 analogues can be elucidated (Figure S7). The resulting fwhm values for all three analogues were of the same order of magnitude, showing that the amount of nickel incorporation does not severely affect the resulting kinetics. Cu-MOF-74 had a fwhm value of 0.84 ± 0.03 min. A slight reduction in the reaction rate is expected when the number of catalytically active available copper sites is reduced, i.e., on nickel incorporation. Surprisingly, the presence of 10 atom % nickel in MOF-74 turned out to be rather beneficial in that the reaction rate of Ni_0.1_Cu_0.9_-MOF-74 was found to be slightly faster (0.73 ± 0.03 min) than that of pure Cu-MOF-74. These faster kinetics follow the same trend observed in the spike of the first GSNO addition, suggesting that GSH binding may be improved by nickel incorporation. However, on further reducing the number of copper sites by incorporating more nickel (25 atom %), the expected reduction in reaction rate was observed, as Ni_0.25_Cu_0.75_-MOF-74 showed a slightly slower reaction rate (0.92 ± 0.08 min) than Cu-MOF-74. This might be caused by the altered material morphology: more intergrown crystallites with fewer or slightly less accessible hexagonal channels. This again suggests that the reaction is not just happening at the crystal surface.

As our previous work showed that Ni-MOF-74 is incapable of producing NO from GSNO, the catalytic production of NO by the Cu/Ni-MOF-74 analogues can only be assigned to the copper sites within the structure.^39^ Furthermore, with additional aliquots of GSNO, the ability of the Cu/Ni-MOF-74 analogues to generate NO is not diminished, highlighting the catalytic behavior of the system.

#### Two-Step NO Delivery

To assess the capability of the mixed metal MOF-74 analogues to both release preadsorbed NO and catalytically generate it from GSNO, all three analogues were activated and loaded with NO and then fully submerged into PBS with a constant N_2_ stream flowing to the detector in the NO analyzer. The results from this combined delivery protocol are listed in Figure 4c. The profiles show for all MOF-74 analogues that the initial release of NO was very rapid, as shown by the near vertical profile. This was not surprising given the samples had been plunged into PBS (100% RH), allowing the quick release of the NO chemisorbed to the metal sites. Even though the rate of release was faster in the higher RH compared to 11% RH (Figure 4a), the magnitude of release was similar, suggesting that moisture ingression is not a limiting factor for the total release of NO. When the quantity of NO fell below ∼5 ppb, GSNO was added in aliquots of 10 μM concentration. As observed previously, all three MOF-74 analogues were able to catalytically generate NO from GSNO (Figure 4c). Analyzing the fwhm values for the spikes from catalytically generated NO (Figure S7) revealed that the two mixed metal MOF-74 analogues had similar release rates to the generation without prior preadsorbed NO loading/release. The Cu-MOF-74 sample showed faster kinetics with a fwhm value of 0.62 ± 0.02 min. This change in release kinetics may be due to the release of copper ions during the partial degradation of this MOF to Cu_3_PO_4_·3H_2_O as the sample was soaked in PBS for 2 h prior to GSNO addition (see subsequent material stability studies).

These NO delivery studies highlight the availability of both the nickel and copper sites within the structure to perform different functions. These Cu/Ni-MOF-74 analogues are capable of a high initial burst release of NO followed by a continuously catalytically generated quantity of NO. This dual-action mechanism demonstrates the versatility and applicability of this MOF platform, as well as reticular chemistry, and may have utility in antimicrobial applications.^46^

### Material Stability

**Figure 5:**
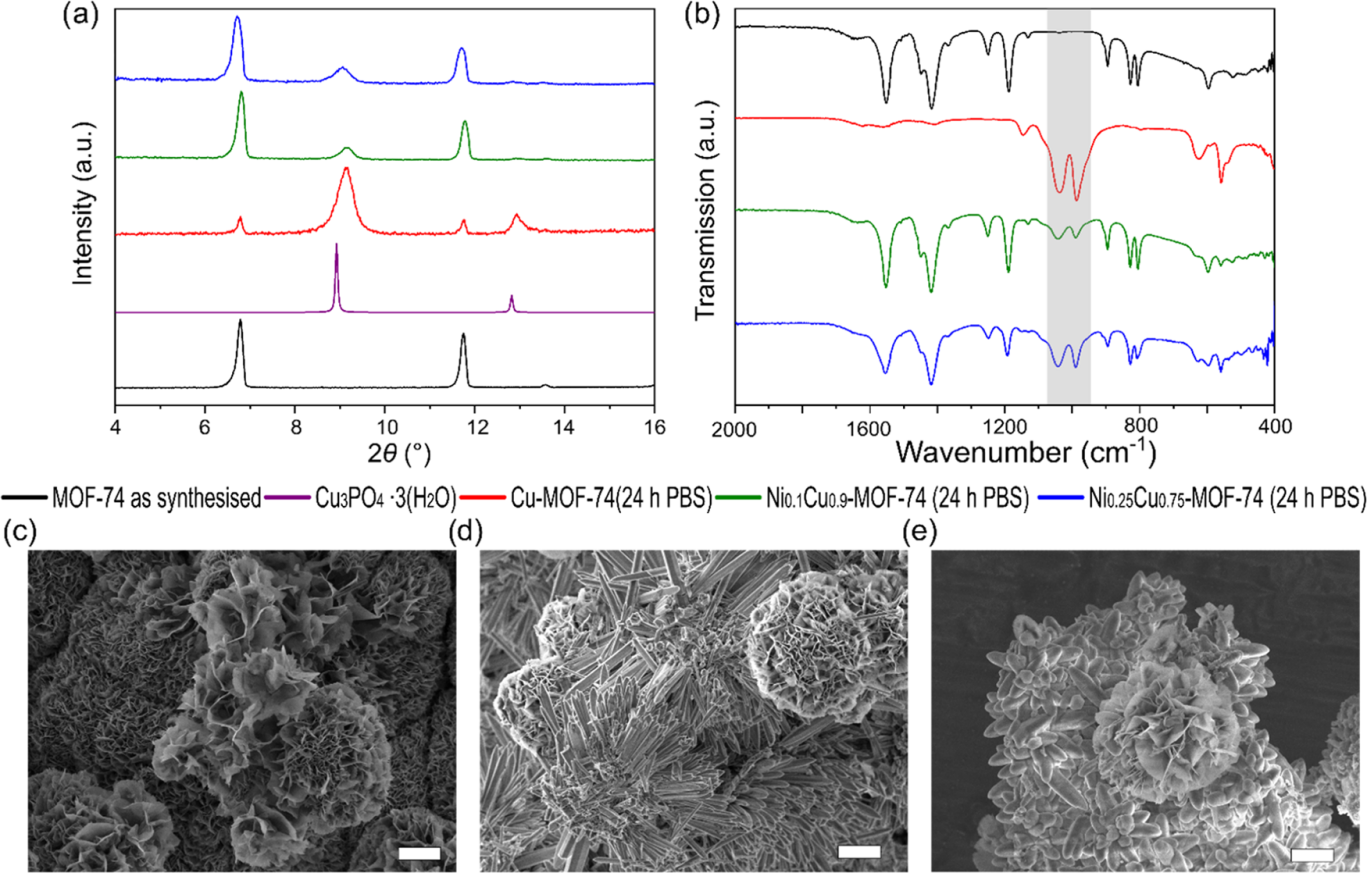
Characterization of Cu/Ni-MOF-74 analogues after immersion in PBS at 37 °C for 24 h. (a) PXRD patterns of Cu-MOF-74 (red), Ni_0.1_Cu_0.9_-MOF-74 (green), and Ni_0.25_Cu_0.75_-MOF-74 (blue), with reference patterns for Cu-MOF-74 (black) and Cu_3_PO_4_·3H_2_O (purple, ICDD: 00-022-0548) taken with Cu–Kα radiation. (b) FTIR spectra of Cu-MOF-74 (red), Ni_0.1_Cu_0.9_-MOF-74 (green), and Ni_0.25_Cu_0.75_-MOF-74 (blue), with reference spectra of Cu-MOF-74 (black) and phosphate peaks highlighted (gray); the SEM image of (c) Cu-MOF-74 (red), (d) Ni_0.1_Cu_0.9_-MOF-74 (green), and (e) Ni_0.25_Cu_0.75_-MOF-74 (blue), scale bar is 5 μm.

After loading and releasing NO in 11% RH, the Cu-MOF-74 maintained its crystallinity and purity, as shown by PXRD (Figure S9). However, after immersion in PBS over the duration of the NO release measurement, the PXRD pattern of the recovered material revealed that there was a reduction in the crystallinity of Cu-MOF-74 and growth of Cu_3_PO_4_·3H_2_O. In light of these findings, we carried out a more comprehensive evaluation of the stability of these Cu/Ni-MOF-74 analogues. The sample materials were soaked in a PBS solution at pH 7.4 at 37 °C from 1 h to 1 week. The powders were recovered and characterized using PXRD, FTIR, and SEM imaging (Figures 5 and S10–S16). The PXRD patterns of Cu-MOF-74 show that there is significant degradation of the structure, resulting in the formation of Cu_3_PO_4_·3H_2_O within 6 h and with almost complete transformation within 24 h (Figures 5a and S11). A similar trend is observed in the FTIR spectra, with phosphate bands appearing in the spectrum at 1 h and becoming dominant after 24 h (Figure 5b, red, and Figure S14). The recorded SEM images (Figures 5c–e and S10) suggest that the Cu_3_PO_4_·3H_2_O forms on the surface of the material and continuously grows outward from these nucleation points. By adding 10 atom % nickel, the stability of Ni_0.1_Cu_0.9_-MOF-74 is dramatically increased; even after 24 h, there is a large proportion of the MOF-74 framework remaining, as shown by the PXRD patterns and FTIR spectra (Figures 5a,b, S12, and S15). The Ni_0.25_Cu_0.75_-MOF-74 has even longer-term stability, as the MOF-74 structure is still the main component after soaking for 1 week in PBS, as can be seen in the PXRD patterns and FTIR spectra (Figures S13 and S16). SEM imaging (Figure S10) shows that there is still a growth of Cu_3_PO_4_·3H_2_O on the surface of both materials. However, this growth is slower as more Ni is added. As the time that these samples release gaseous NO is within 3 h, the increased stability provided by 10 atom % nickel will significantly reduce the relevant toxicity of the material. Furthermore, if formulated, for example, in a polymer, we would expect the stability of these materials to be prolonged.^46^ The enhanced stability observed with the incorporation of nickel may be attributed to the fact that nickel exhibits greater inertness compared to copper,^38^ and the stabilizing effect of nickel in different MOF-74 frameworks has been reported previously.^47,48^

The Cu_3_PO_4_·3H_2_O produced from Cu-MOF-74 after a week in PBS at 37 °C was tested with respect to its ability to catalytically generate NO from GSNO. Overall, the copper sites in Cu_3_PO_4_·3H_2_O remain active for oxidizing GSNO, albeit the complete breakdown of GSNO takes more than 10 min, making the process approximately half as fast as that observed with the parent MOF (Figure S8). This indicates that the formation of some Cu_3_PO_4_·3H_2_O is not detrimental to the overall delivery of NO from these MOF-74 analogues.

## Conclusions

In this study, we show how nickel can be successfully incorporated into Cu-MOF-74, with loading levels of up to 25 atom %. Higher amounts of nickel lead to a decrease in the available surface area due to the intergrowth of crystallites and the formation of nanosized Cu-rich agglomerates. Adding only a small amount of nickel (10 atom %) can produce more than a 7-fold increase in deliverable NO storage while the same catalytic ability to generate NO from GSNO is retained. Furthermore, the introduction of nickel to the framework enhances the materials’ stability toward phosphates, which is an important parameter for potential use in medicinal applications (Table 1). Ongoing development efforts are focused on integrating these MOF-74 analogues into polymer membranes in order to produce a simple composite material capable of dual-action NO release behavior that may be applicable in biomedicine.

**Table 1:** Table Outlining the Relative Merits of the Three Cu/Ni-MOF-74 Analogues

MOF-74 analogue	material homogeneity	moisture-triggered NO release	catalytic NO generation	stability in PBS (pH 7.4) after 24 h
Cu-MOF-74	√	×	√	×
Ni_0.1_Cu_0.9_-MOF-74	√	√	√	√
Ni_0.25_Cu_0.75_-MOF-74	×	√	√	√

In summary, the Ni_0.1_Cu_0.9_-MOF-74 structure presented here shows several excellent properties that make it highly suitable for use in the next generation of NO delivery devices. It is capable of both burst release and controlled, continuous generation of NO under biological conditions while demonstrating high thermal and chemical stability in physiological media. This study further highlights the power of reticular chemistry and how the variable functionality of MOFs makes them extremely well-suited to creating complex multifunctional materials.

## Methods

### Synthesis of Cu-MOF-74

Copper(II) nitrate trihydrate (2.5 mmol), 2,5-Dihydroxyterephthalic acid (1.25 mmol), and 1,4-diazabicyclo[2.2.2]octane (0.625 mmol) were dissolved in 15 mL of DMF. The solution was placed in a Teflon-lined autoclave and heated to 60 °C for 3 days. The resultant product was separated via filtration, washed thoroughly with DMF, water, ethanol, and methanol, and dried overnight at 60 °C.

### Synthesis of Ni_0.1_Cu_0.9_-MOF-74

Copper(II) nitrate trihydrate (1.25 mmol) and nickel(II) nitrate trihydrate (1.25 mmol) were each dissolved separately in 5 mL of DMF. 2,5-dihydroxyterephthalic acid (1.25 mmol) and 1,4-diazabicyclo[2.2.2]octane (0.625 mmol) were dissolved in a further 5 mL of DMF. The solutions were mixed and placed in a Teflon-lined autoclave and heated to 60 °C for 3 days. The resultant product was separated via filtration, washed thoroughly with DMF, water, ethanol, and methanol, and dried overnight at 60 °C.

### Synthesis of Ni_0.25_Cu_0.75_-MOF-74

Copper(II) nitrate trihydrate (0.625 mmol), nickel(II) nitrate trihydrate (1.875 mmol), 2,5-dihydroxyterephthalic acid (1.25 mmol), and 1,4-diazabicyclo[2.2.2]octane (0.625 mmol) were dissolved in 15 mL of DMF. The solution was placed in a Teflon-lined autoclave and heated to 60 °C for 3 days. The resultant product was separated via filtration, washed thoroughly with DMF, water, ethanol, and methanol, and dried overnight at 60 °C.

All syntheses were repeated to ensure reproducibility.

### NO Loading and Release

Activation and NO loading were performed using a similar protocol as previously reported.^39^ 10–20 mg of sample was placed in a 5 mL glass vial inside a Schlenk tube and activated for 16 h at 150 °C under a reduced pressure of 10^–3^ mbar. This thermal treatment removes occluded solvent molecules and creates open metal sites in the framework. After activation, the samples were introduced to a NO atmosphere (2 bar absolute pressure) for 1 h, after which the remaining NO was replaced with argon, and the samples were sealed under dry Ar. To measure the release of NO from the MOFs, samples were placed into a chamber, whereby a flow of moist N2 gas (11% RH) was passed over the sample. The release of the gaseous NO was recorded via a chemiluminescence reaction with ozone using a nitric oxide analyzer (NOA) (280i, Zysense, Weddington, North Carolina). Kinetic release measurements were recorded within 1 day of NO loading. Data acquisition was stopped once the release levels fell below ∼10 ppb. Each measurement was recorded in duplicate, and the resulting NO release was calculated as a function of time.

### Catalytic Generation of NO

As-synthesized MOF samples were placed and sealed in a septum-topped vial, the reaction cell. An inlet flow of N2 gas, along with an outlet flow, was hooked up to the NOA. After initiating data collection on the NOA, the MOF samples were soaked in 3 mL of phosphate-buffered saline (PBS, pH = 7.4) in the reaction cell. GSNO was then injected, yielding a concentration of 10 μM. The reaction was shielded from light using aluminum foil and measured at room temperature (approximately 20 °C) for 1 h with a collection interval of 1 s. Once the signal fell below ∼5 ppb, fresh PBS was injected to ensure all NO was released from the MOF. The GSNO addition process was repeated twice more. In some repeats, when the signal plateaued on the baseline level signal, 10 μL of 0.1 M CuCl_2_ was added to force the release of NO from any GSNO remaining in the reaction cell. Each measurement was recorded in duplicate, and the resulting NO release was plotted against time.

### Dual NO Delivery

Both previously described measurement protocols were combined. The MOFs were activated for 16 h at 150 °C under a reduced pressure of 10^–3^ mbar. The samples were then introduced to a NO atmosphere (2 bar absolute pressure) for 1 h; excess NO was evacuated and backfilled with dry argon. To record the initial kinetic NO release profile, 3 mL of PBS (pH = 7.4) was added to the MOF and data was acquired with a collection interval of 1 s. When the NO release measured fell below ∼5 ppb, another 3 mL of PBS and 10 μM GSNO were injected into the reaction cell. The sample was shielded from light using aluminum foil, and measurements were recorded at room temperature (approximately 20 °C) for another 1 h. Each measurement was recorded in duplicate, and the resulting NO release was plotted against time.

### PBS Stability Study

Four samples of each material (≈3.5 mg) were placed in centrifuge tubes containing 15 mL of PBS (pH 7.4). The samples were kept at 37 °C for 1, 6, 24, and 1 week. Samples were centrifuged to recover the material, dried, and characterized.

### Characterization Techniques

Powder X-ray diffraction (PXRD) patterns were recorded on a STOE STADI/P diffractometer using Mo Kα1 radiation at room temperature in capillary Debye–Scherrer mode. The samples from the PBS stability study were measured using a PANalytical Empyrean diffractometer using Cu Kα1 radiation at room temperature in reflection, Bragg–Brentano, Theta −2θ mode. FTIR spectroscopy was carried out using a Shimadzu IR Affinity-1 FTIR spectrophotometer in transmittance mode from 400 to 4000 cm^–1^. Thermogravimetric analyses (TGA) were recorded in air on a Stanton Redcroft STA-780 from room temperature to 450 °C, with a heating rate of 5 °C/min. N_2_ adsorption isotherms were recorded on a Micromeritics Tristar ii Surface Area and Porosity Instrument. Samples were added to a frit tube and activated *in vacuo* (150 °C, ∼3 × 10^–5^ mbar, 16 h) prior to the measurement. SEM micrographs were collected using a JEOL IT800 at a working distance of 4 mm and low operating voltages (2–5 kV) to ensure sensitive mapping of the surface. The powder samples were placed on aluminum tape. ICP-OES analyses were performed on a PerkinElmer Optima 5300DV instrument with samples digested in HNO_3_ and then diluted. TEM micrographs were obtained using an FEI Titan Themis operated at 200 kV on samples prepared by deposition of one drop of the nanoparticle suspension onto holey carbon films supported on a 300 mesh Cu grid (Agar Scientific). NO release measurements were recorded on a nitric oxide analyzer (NOA, 280i, Sievers) using the chemiluminescence technique. All measurements for kinetic release were run in duplicates.

## Data Availability

The research data supporting this publication can be accessed at https://doi.org/10.17630/b51e1007-e212-49b3-98e1-6212c3d5e54d.

## Supporting Information Available

The Supporting Information is available free of charge at https://pubs.acs.org/doi/10.1021/acs.inorgchem.4c05125. The research data supporting this publication can be accessed at https://doi.org/10.17630/b51e1007-e212-49b3-98e1-6212c3d5e54d.

FTIR spectra, TGA data, and N_2_ adsorption isotherms of each MOF-74 analogue presented; NO release profile of PBS without MOF after GSNO and subsequent CuCl_2_ additions; Plot showing fwhm data on NO generation from GSNO for all three analogues NO release with and without previous NO release; NO release profile of Cu-MOF-74 after 1 week in PBS (Cu_3_PO_4_·3H_2_O) SEM, FTIR and PXRD data of all three MOF-74 analogues after 1 h, 6 h, 24 h, and 1 week in PBS at 37 °C. (PDF)
