# Supplementary material for: Dual-Action NO Delivery from One Mixed Metal Metal–Organic Framework
Source: Inorg Chem. 2025 Feb 5;64(9):4399–407. doi: 10.1021/acs.inorgchem.4c05125 (PMC11898063; doi:10.1021/acs.inorgchem.4c05125)
Supplement: Supplementary file 1 — ic4c05125_si_001.pdf [file ic4c05125_si_001.pdf]

Supplementary Information

Dual Action NO Delivery from One Mixed Metal Metal-Organic Framework

Russell M. Main, [1*] Aaron B. Naden,[1] Morven J. Duncan,[1] Russell E. Morris,[1] and Romy Ettlinger [1,2*]

[1] EaStCHEM School of Chemistry, Purdie Building, North Haugh, St Andrews KY16 9ST, United Kingdom

[2] TUM School of Natural Sciences, Technical University of Munich, Lichtenbergstrasse 4, 85748 Garching, Germany

E-mail: romy.ettlinger@tum.de

Contents

Contents 1
1. Materials Characterization 2
2. NO Delivery Studies 6
3. PBS stability studies 9
3.1. SEM Imaging 9
3.2. PXRD Analysis 10
3.3. FTIR Analysis 12

1. Materials Characterization

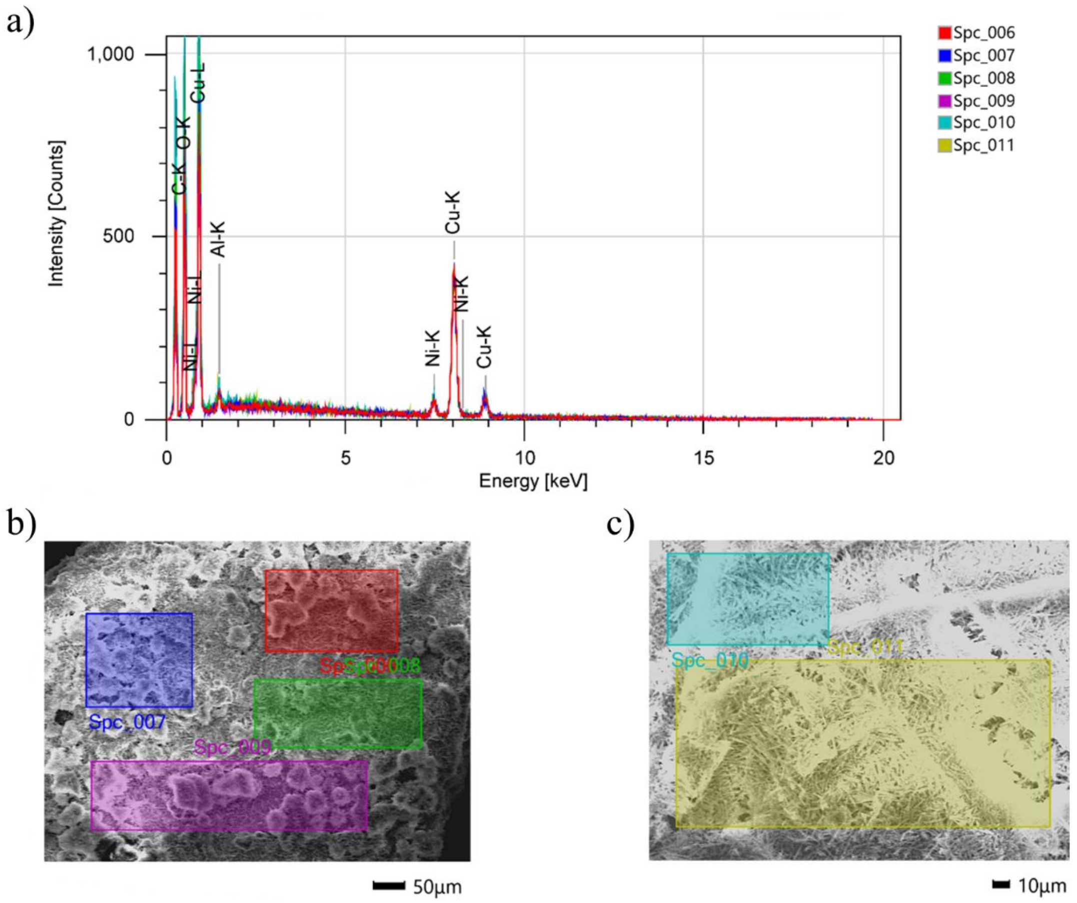

Figure S-1: EDX (a) spectra and (b, c) images with the regions of interest annotated from $Ni_{0.1}Cu_{0.9}$-MOF-74, with Al tape being the support.

Table S-1: The Ni and Cu atom% in $Ni_{0.1}Cu_{0.9}$-MOF-74 as calculated from EDX analysis.

Name	Ni in at%	Cu in at%
Spc 006	8.04	91.96
Spc 007	8.55	91.45
Spc 008	7.95	92.05
Spc 009	8.25	91.75
Spc 010	8.17	91.83
Spc 011	7.48	92.52
Average	8.07±0.33	91.93±0.33

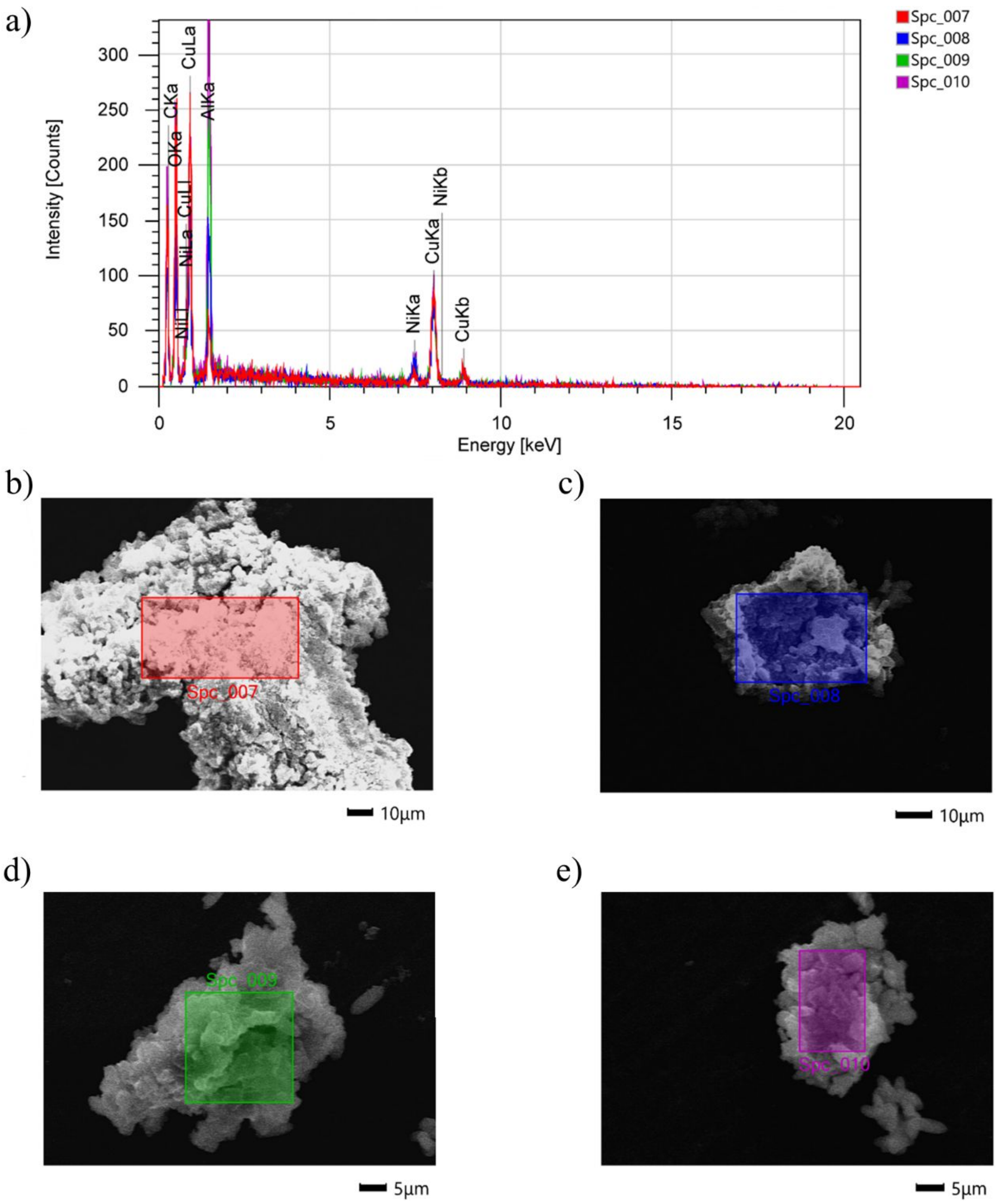

Figure S-2: EDX (a) spectra and (b, c, d, e) images with the regions of interest annotated from $Ni_{0.25}Cu_{0.75}$-MOF-74, with Al tape being the support.

Table S-2: The Ni and Cu atom% in as calculated from EDX analysis.

Name	Ni in at%	Cu in at%
Spc 007	22.41	77.59
Spc 008	23.56	76.44
Spc 009	25.81	74.19
Spc 010	27.54	72.46
Average	24.91±0.51	75.09±0.77

Table S-3: The Ni and Cu concentrations in at% as derived from ICP-OES analysis for $Ni_{0.1}Cu_{0.9}$-MOF-74 and $Ni_{0.25}Cu_{0.75}$-MOF-74 as well as the calculated average at% for the two analogues.

Sample	Ni in at%	Cu in at%
	8.11	91.89
$Ni_{0.1}Cu_{0.9}$-MOF-74	7.97	92.03
	8.06	91.94
Average	8.05±0.06	91.95±0.06
	17.00	83.00
$Ni_{0.25}Cu_{0.75}$-MOF-74	15.82	84.18
	16.86	83.20
Average	16.50±0.52	83.50±0.52

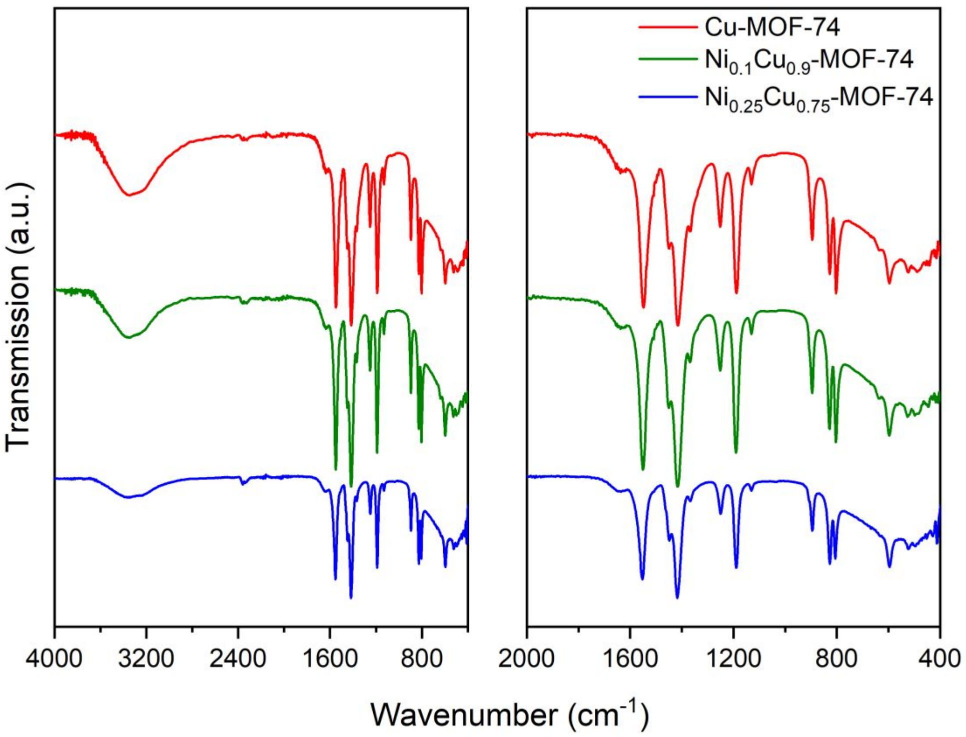

Figure S-3: FTIR spectra of Cu-MOF-74 (red), $Ni_{0.1}Cu_{0.9}$-MOF-74 (green) and $Ni_{0.25}Cu_{0.75}$-MOF-74 (blue).

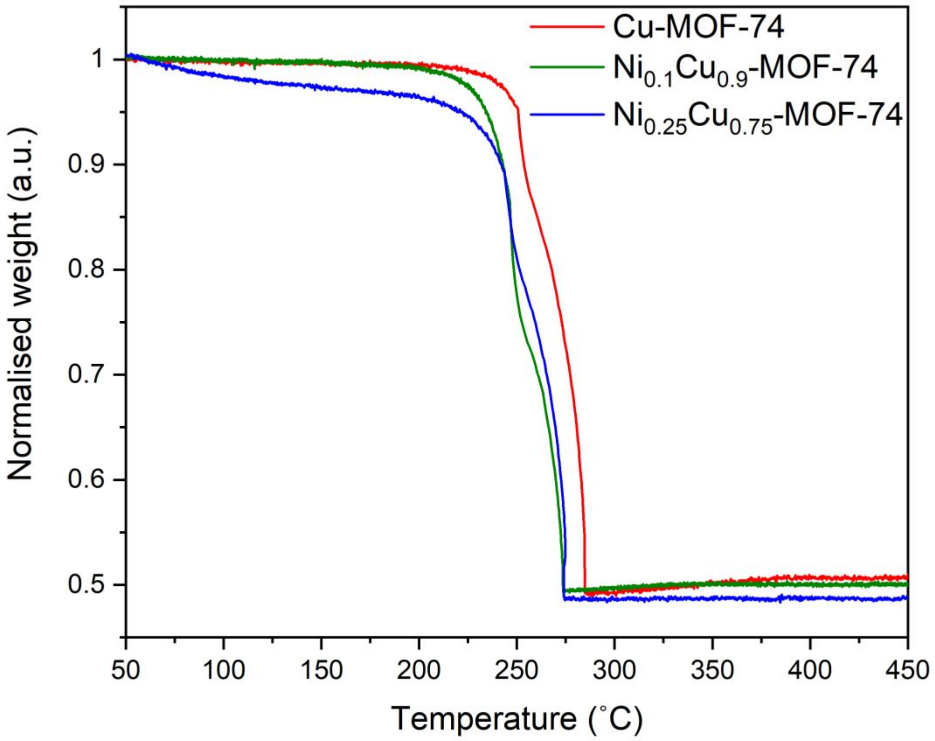

Figure S-4:Thermal gravimetric analysis plots of Cu-MOF-74 (red), $Ni_{0.1}Cu_{0.9}$-MOF-74 (green) and $Ni_{0.25}Cu_{0.75}$-MOF-74 (blue) recorded at 5° C/min in air.

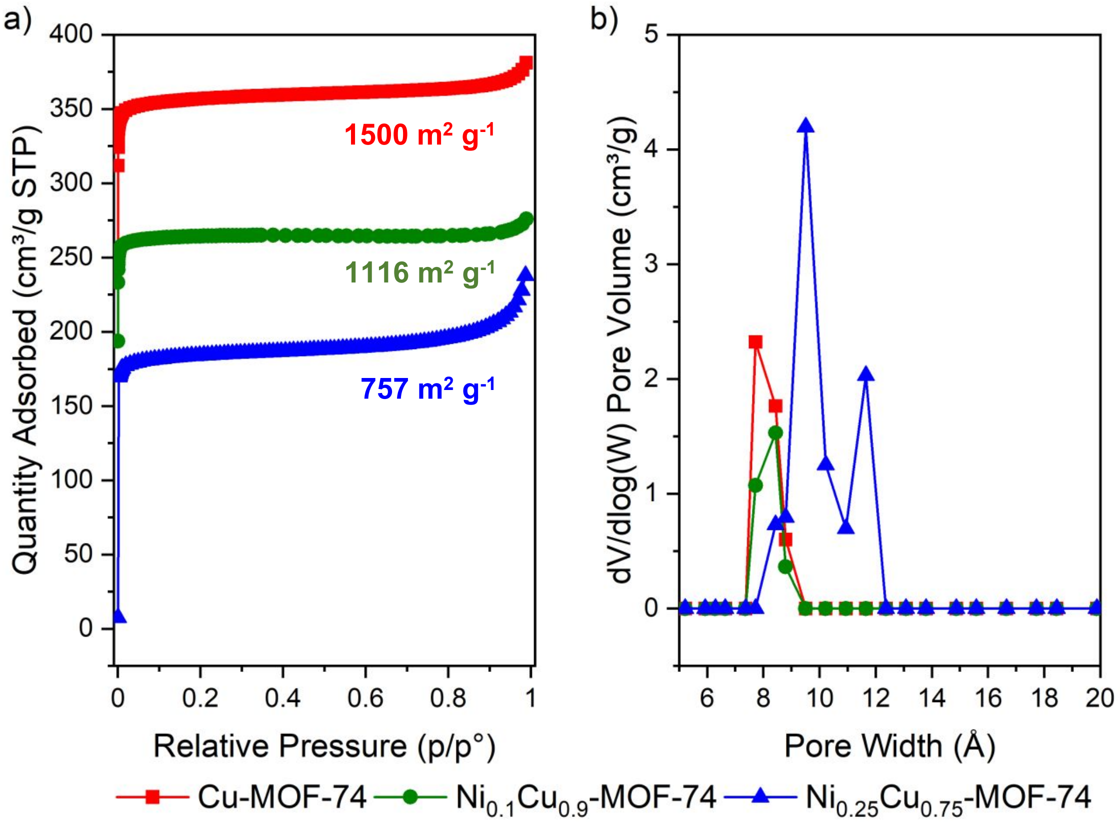

Figure S-5: (a) N_2 adsorption isotherms at 77 K for Cu-MOF-74 (red), $Ni_{0.1}Cu_{0.9}$-MOF-74 (green) and $Ni_{0.25}Cu_{0.75}$-MOF-74 (blue); (b) Pore size distribution plots for Cu-MOF-74 (red), $Ni_{0.1}Cu_{0.9}$-MOF-74 (green) and $Ni_{0.25}Cu_{0.75}$-MOF-74 (blue) using a Tarazona NLDFT model.

2. NO Delivery Studies

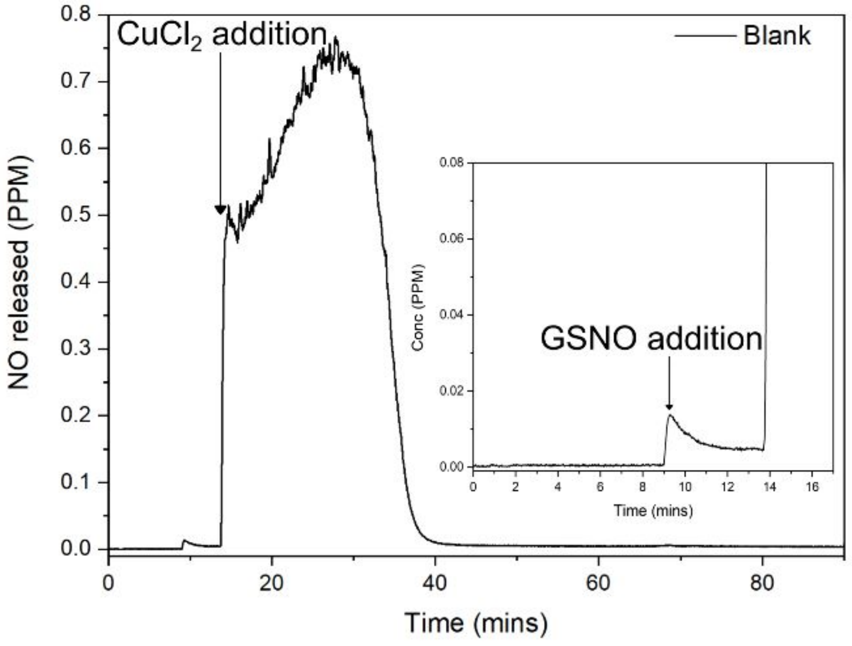

Figure S-6: NO release profile of GSNO addition in PBS with no MOF present followed by CuCl$_2$ addition.

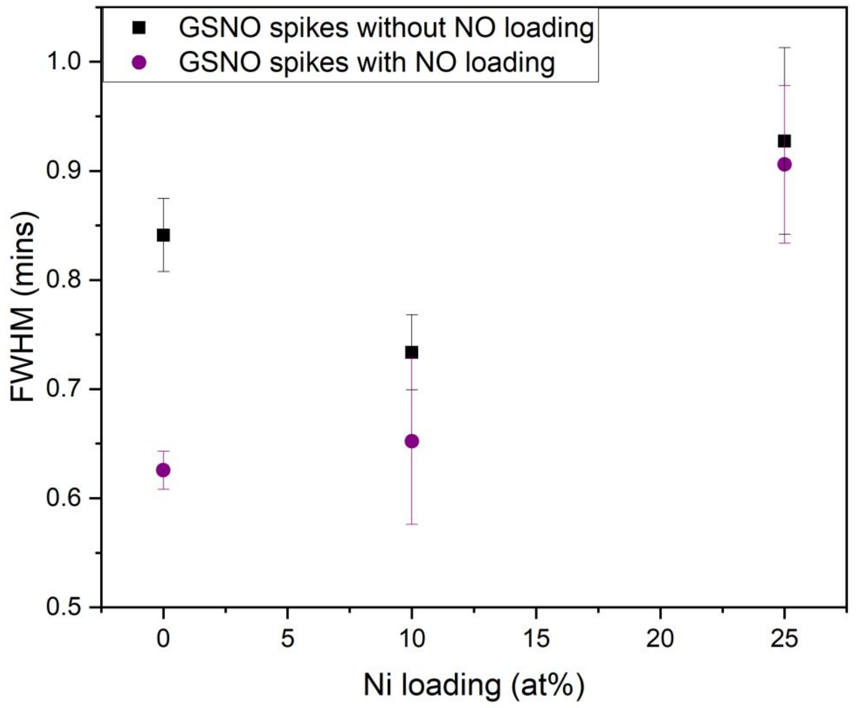

Figure S-7: Plot showing how Ni loading affects peak widths of NO release spike on GSNO addition. Without NO loading (black) and with (purple).

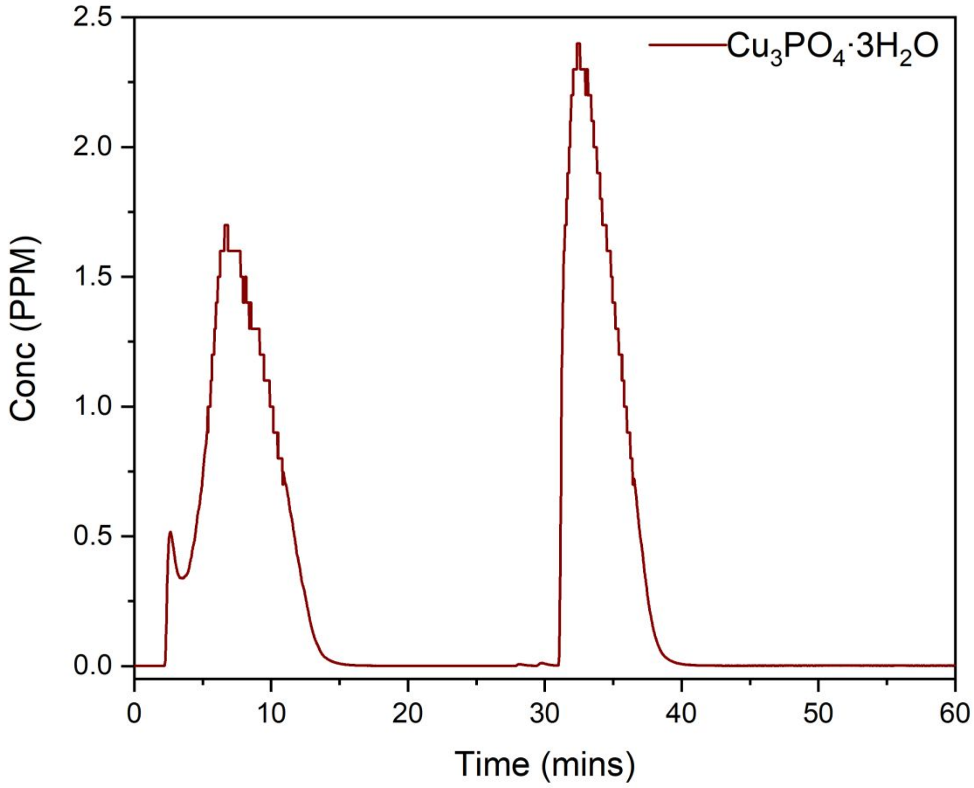

Figure S-8: NO release profile of GSNO addition in PBS containing $Cu_3PO_4·3H_2O$ derived from Cu-MOF-74.

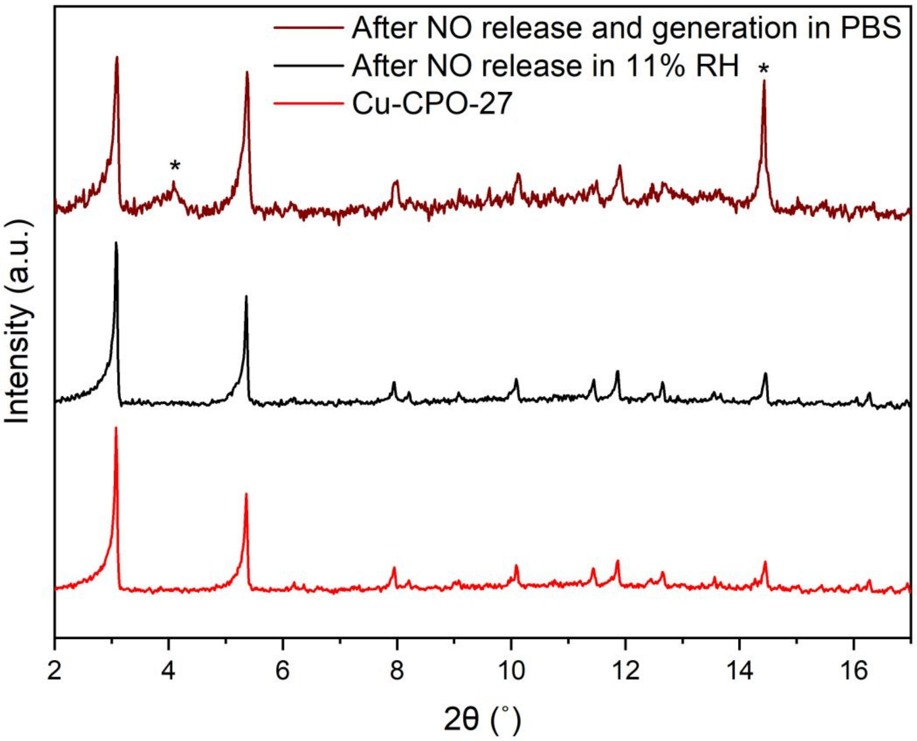

Figure S-9: Intensity normalised PXRD patterns of Cu-CPO-27 (red), after releasing NO in a 11% RH gas flow (black) and after first releasing pre-adsorbed NO and secondly catalytically generating NO from GSNO in PBS (brown). Characteristic peaks of $Cu_3PO_4{\cdot}3H_2O$are marked (*). Patterns taken with Mo-Kα radiation.

3. PBS stability studies

3.1. SEM Imaging

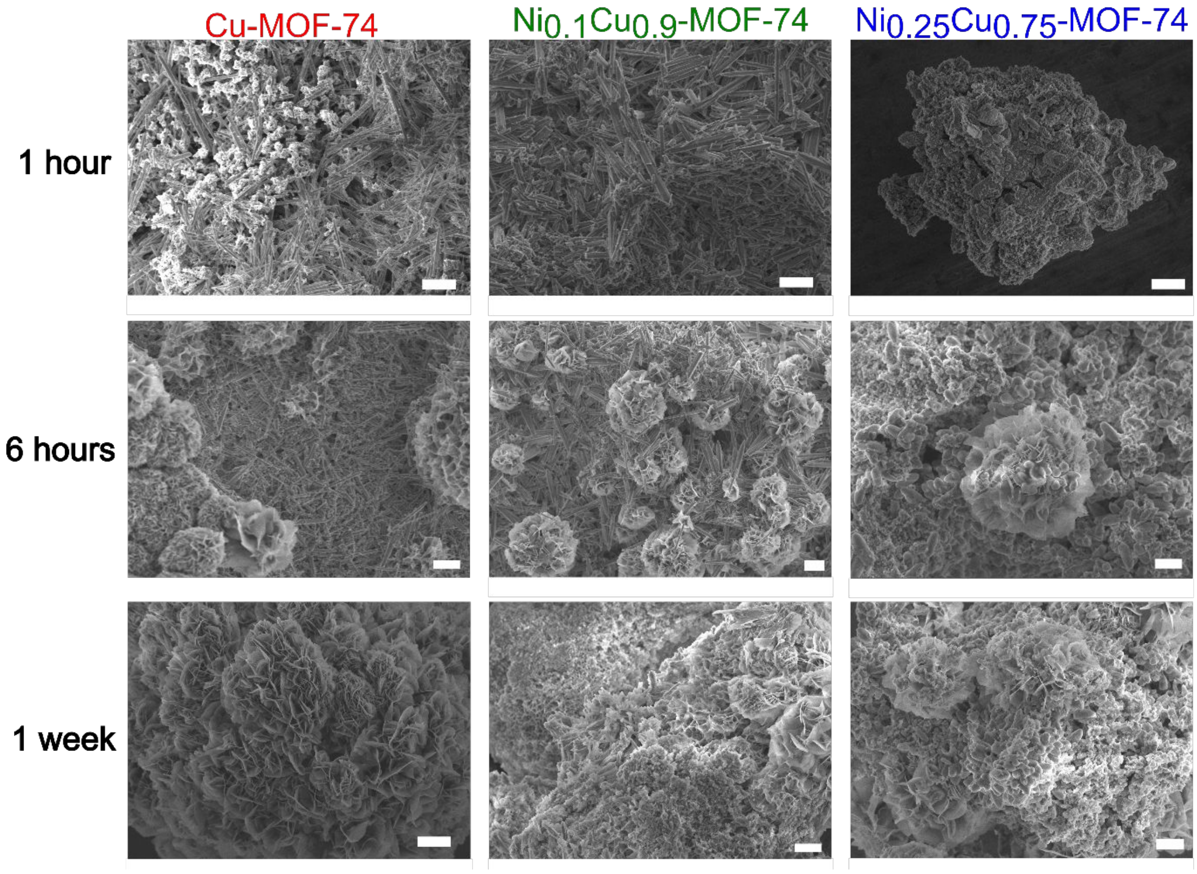

Figure S-10: SEM images of Cu-MOF-74, $Ni_{0.1}Cu_{0.9}$-MOF-74 and $Ni_{0.25}Cu_{0.75}$-MOF-74 after 1 h, 6 h and 1 week in PBS (pH 7.4) at 37 ˚C. The scale bar for all images is 5 μm.

3.2. PXRD Analysis

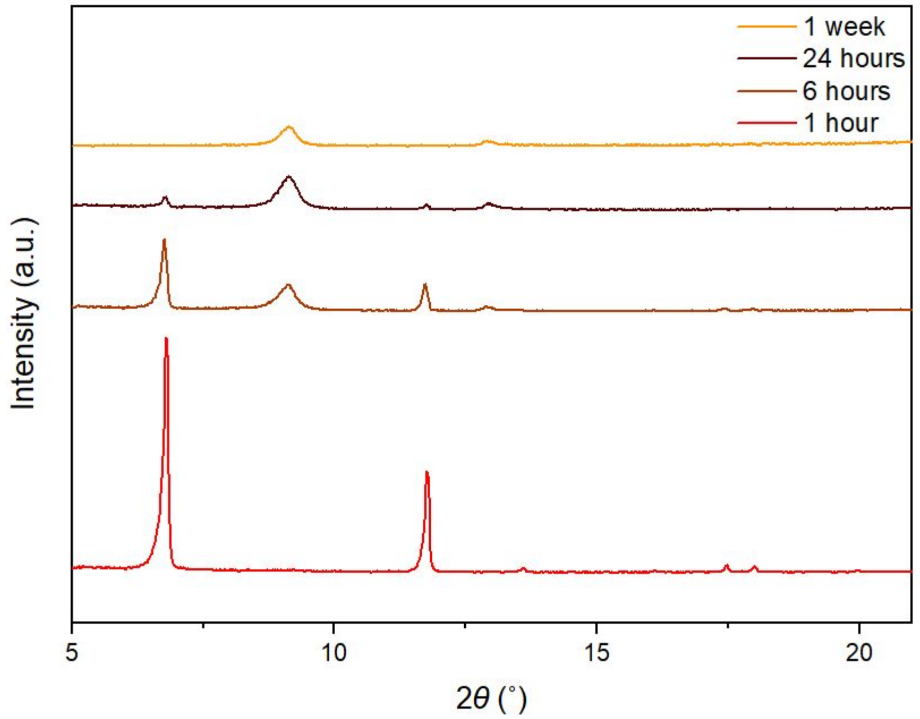

Figure S-11: Intensity normalised PXRD patterns of Cu-MOF-74 after soaking in PBS at 37 ˚C. Recorded using Cu-Kα radiation.

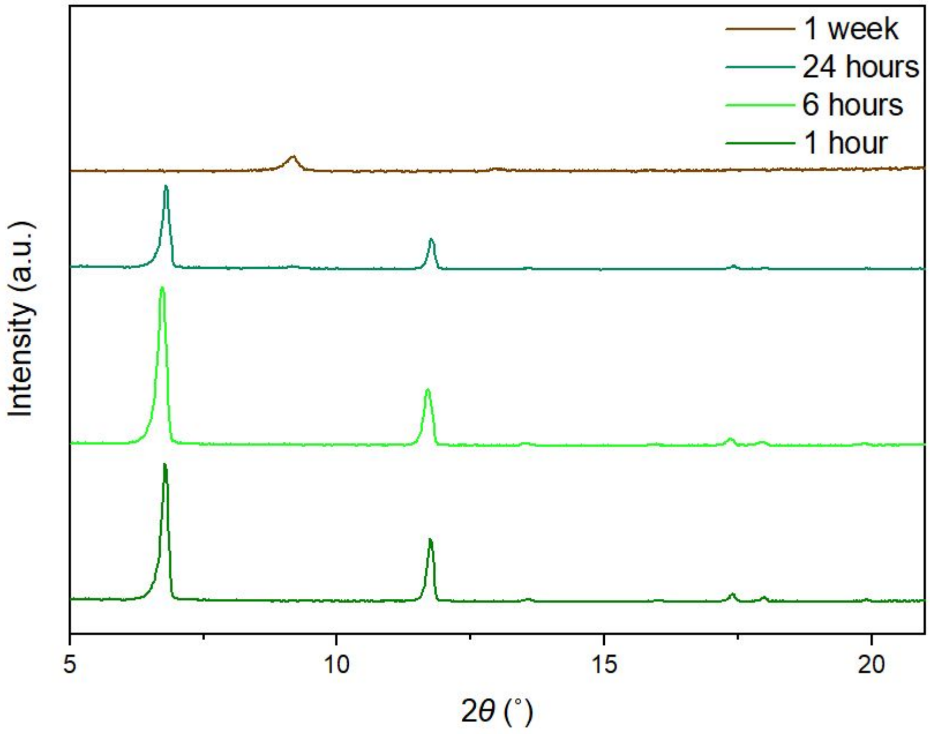

Figure S-12: Intensity normalised PXRD patterns of $Ni_{0.1}Cu_{0.9}$-MOF-74 after soaking in PBS at 37 ˚C. Recorded using Cu-Kα radiation.

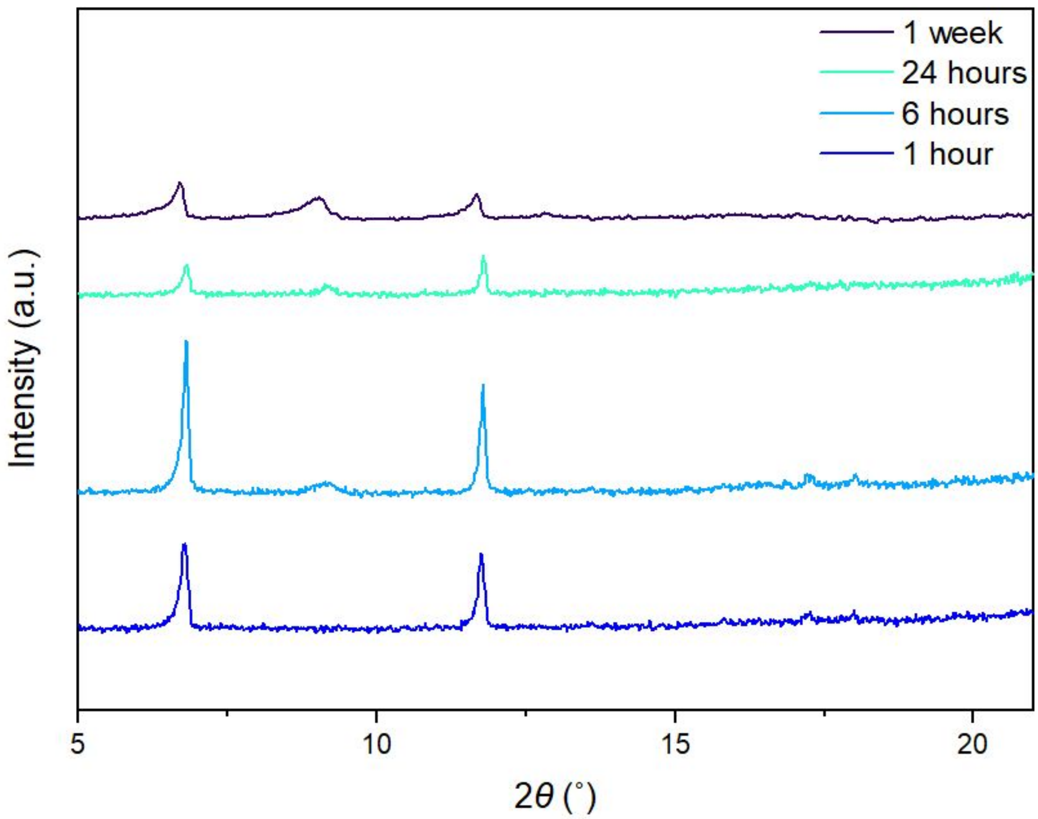

Figure S-13: Intensity normalised PXRD patterns of $Ni_{0.25}Cu_{0.75}$-MOF-74 after soaking in PBS at 37 ˚C. Recorded using Cu-Kα radiation.

3.3. FTIR Analysis

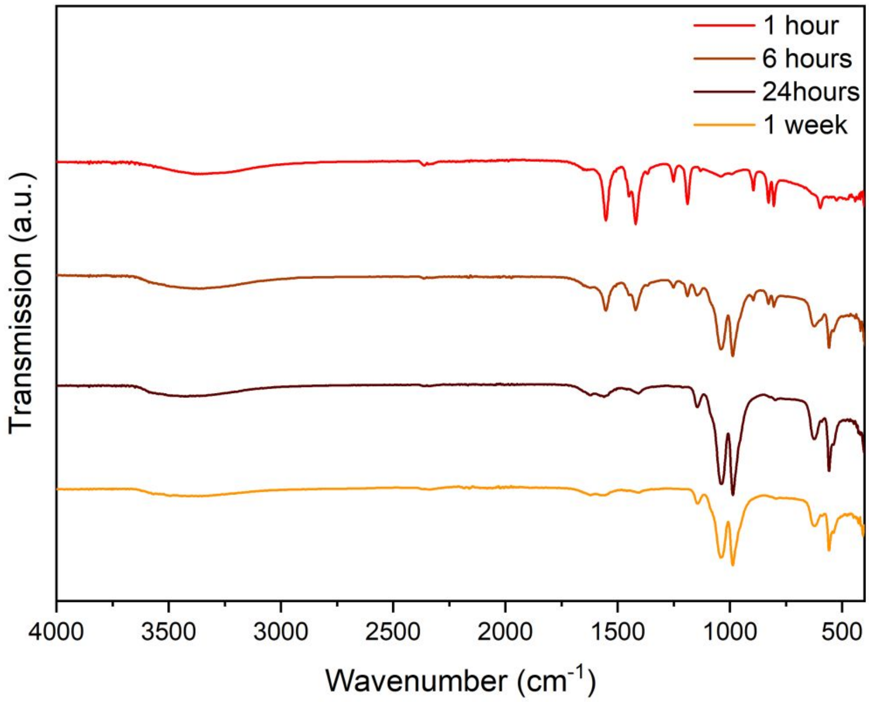

Figure S-14: FTIR spectra of Cu-MOF-74 after soaking in PBS at 37 ˚C.

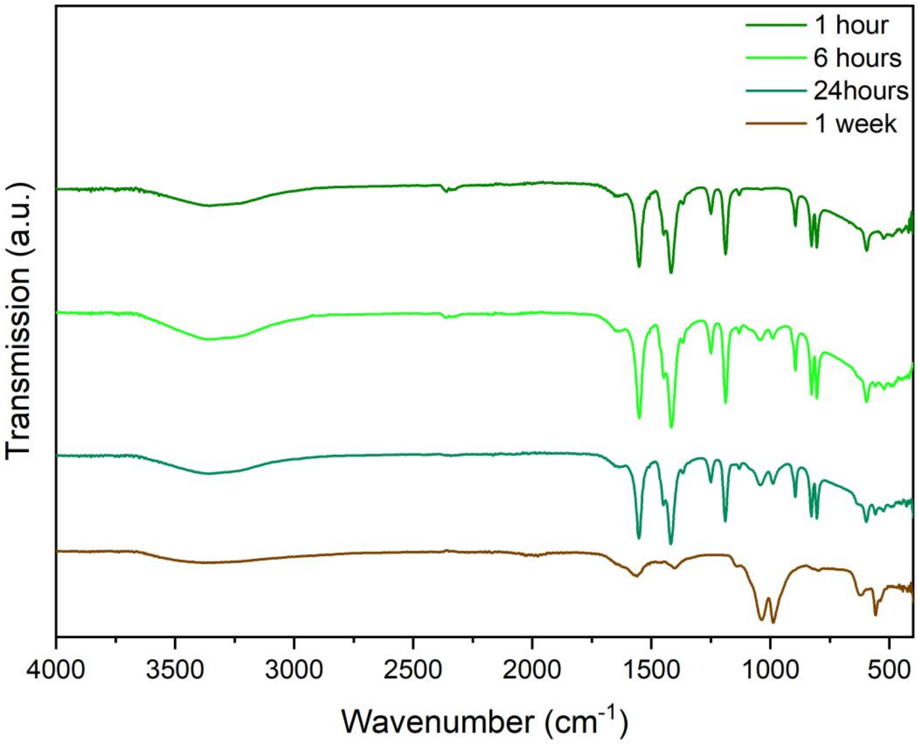

Figure S-15:FTIR spectra of $Ni_{0.1}Cu_{0.9}$-MOF-74 after soaking in PBS at 37 ˚C.

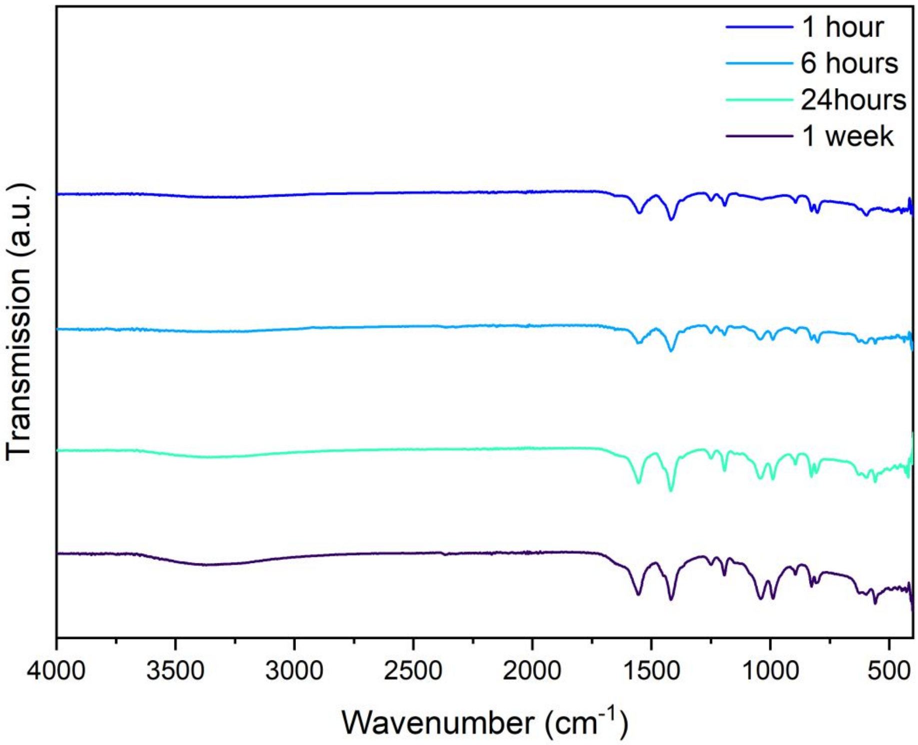

Figure S-16: FTIR spectra of $Ni_{0.25}Cu_{0.75}$-MOF-74 after soaking in PBS at 37 ˚C.
